# Spatio-temporal characteristics and influencing factors of urban shrinkage in county level of Heilongjiang Province, Northeast China

**DOI:** 10.1016/j.heliyon.2023.e21436

**Published:** 2023-10-23

**Authors:** Junqi Huo, Shanlin Huang

**Affiliations:** a College of Public Administration and Law, Northeast Agricultural University, Harbin, 150000, China; b Research Center of Modern Agricultural Development, Northeast Agricultural University, Harbin, 150030, China

**Keywords:** Urban shrinkage, Spatial-temporal characteristics, Influencing factors, County-level cities, Northeast China

## Abstract

Urban shrinkage has become increasingly prevalent in the context of global urbanization. Understanding its spatio-temporal characteristics and influencing factors is of great significance for promoting sustainable development and high-quality urbanization. This paper identified county-level shrinking cities in Heilongjiang Province from the perspective of urban entities. It employed spatial autocorrelation methods to analyze their spatial-temporal characteristics and explored the factors that contributed to this phenomenon. The results indicated that between 2010 and 2020, the phenomenon of urban shrinkage at the county level in Heilongjiang Province was primarily concentrated in the province's hinterland. Moreover, they exhibited characteristics of the Great Recession and Small Growth. The main factors influencing the shrinkage included the urban industrial structure, living standards, and population composition.

## 1 Introduction

Since the last century, cities in many countries have been expanding [[1], [2], [3]], and a large number of people have moved to cities. The rapid urbanization process seems to cover up the fact that cities are not capable of infinite growth. In 1988, Häußermann and Siebel first proposed “urban shrinkage” in their study of urban population loss in Germany [4]. The phenomenon of urban population loss due to factors such as deindustrialization and ageing. In the context of global urbanization, urban shrinkage in local areas is beginning to be focused on [5]. Currently, a large number of cities in Europe, the United States and other countries have experienced urban shrinkage [6,7]. And some cities in China have also experienced population loss [8], it is not limited to underdeveloped areas [9]. Relatively developed city clusters also shows different degree of urban shrinkage [10]. Although “urban shrinkage” is not equivalent to “urban recession” [11], it may still lead to economic and social problems such as housing vacancy, economic vitality and spatial quality decline [[12], [13], [14], [15], [16], [17]]. Therefore, in the context of building sustainable cities in the United Nations 2030 Sustainable Development Goals, the characteristics and causes of urban shrinkage need to be explored urgently in order to serve as a guide for urbanization.

For urban shrinkage research, the first thing to consider is how to accurately identify urban shrinkage. At present, some scholars believe that the study of urban shrinkage in China is not standardized, because the calibre of statistics is often the extent of the administrative region. In China, the administrative district of a city may contain non-urban areas such as the county and rural areas under its jurisdiction. This leads to recognition that the results are not scientific, when the county seat of a city shrinks, or in the case of shrinkage in the countryside within its administrative area. The City Entity itself may still be growing, but statistics show that cities are shrinking. This leads to a blind widening of the identification results and a preference for identifying “shrinking areas” rather than “shrinking cities” [18].Therefore, this paper aims to reveal the spatial-temporal characteristics and influencing factors of county-level urban shrinkage in Heilongjiang Province from 2010 to 2020 under the perspective of urban entities, so as to contribute Chinese sample to the studies of urban shrinkage. Based on “two-step diagnostic method” for urban shrinkage [16], after the initial identification of urban shrinkage through population change, this paper replaces the city administrative boundary with the boundary of the city built-up area and extracts the nighttime lighting data of the city entity to identify the shrinking city, which excludes disturbances in non-urban areas and makes the results more internationally comparable. In addition, urban shrinkage is a continuous and long-lasting process in which some cities have an increasing trend in the shrinkage process, but if not studied in a continuous time series, it may not be possible to fully reveal their timing characteristics due to the choice of time span of the study. So, the cross-sensor corrected “NPP-VIIRS-like NTL Data” [19] was used to overcome the differences of different nighttime lighting data sets to achieve a long-term span study so as to fully reveal the influencing factors of county-level city shrinkage in Heilongjiang Province in a consecutive ten-years time series.

Based on the above two points, this paper can reveal its temporal and spatial characteristics with more scientific and realistic urban shrinkage recognition results, and reveal its influencing factors in long-term sequences. On the one hand, it can provide reference for improving the more scientific method of urban shrinkage recognition. On the other hand, it can also provide more realistic policy advice to local governments and provide a reference for Heilongjiang province and other provinces and countries to deal with urban shrinkage.

The remaining structure of this paper is as follows: The second part is a review of the relevant literature, the third part introduces the research area, research methods and data sources, the fourth part is the empirical analysis results, the fifth part is a discussion, the sixth part is a conclusion and policy recommendations.

## 2 Literature review

Literatures focus on urban shrinkage are now enriched. In some studies, a single index of population change was used to identify urban shrinkage [20,21]. However, due to the lack of consensus on the quantitative identification criteria of urban shrinkage, different studies have different criteria for identifying urban shrinkage based on population change. It is reasonable to identify urban shrinkage by the rate of population change [20], the degree of population loss in successive years [22] and the scale of population loss on the long scale [23]. However, urban shrinkage is complex, which is not only manifested by population loss, but also contains a series of socio-economic characteristics [24]. For example, in Shrinking City International Research Network's definition of shrinking city, in addition to population loss, structural economic transformation and crisis were mentioned [25]. However, urban economic downturn tends to lag behind population loss [17], and in the development process of some cities, low-end labor force outflow was caused by the upgrading of industrial structure. It is obviously questionable whether this phenomenon can be simply defined as urban shrinkage by population change [26].

Therefore, some studies began to take more comprehensive consideration of urban shrinkage, including economic [27], social [28] and spatial [29] factors in the identification of urban shrinkage, and it has been found to coexist with population loss and economic growth in some cities [28]. Therefore, although population change is an important cause of urban shrinkage [6], identifying urban shrinkage only with the indicator of population change may expand the identification result. It is also necessary to comprehensively consider urban shrinkage from social, economic and other multi-dimensional perspectives. Nighttime light data can accurately reflect the intensity of economic activities in human society [30], and has been widely used in studies to assess the urban population size, economic development level and extract the urban built-up areas [31,32]. In addition, nighttime light data can more objectively and sensitively reflect whether a city shrunk [33], so it is also widely used in the studies of urban shrinkage [[34], [35], [36]]. However, due to the significant differences between the nighttime light data of NPP-VIIRS from 2012 to present and DMSP-OLS as of 2013 in terms of sensor parameters and data quality and other aspects [37], the research time span for identifying urban shrinkage based on nighttime light data was limited.

While the urban shrinkage identification method is constantly improving, the research topic of urban shrinkage is no longer limited to the identification of the shrinkage phenomenon. It starts to explore the spatial-temporal characteristics and causes of urban shrinkage phenomenon at the national, regional and provincial scales [9,[37], [38], [39]]. However, the research objects are mainly prefecture-level cities. In recent years, the Chinese government has attached great importance to the development of county-level cities. In 2020, the Chinese government issued the document of Opinions on Promoting the Urbanization Construction with County Towns as the Important Carrier, aiming to promote the high-quality urbanization of county-level cities. But, there are still few researches on the factors that contribute to urban shrinkage in county-level cities, especially in Heilongjiang Province [40] where urban shrinkage is relatively concentrated in China. In addition, it should be noted that “urban entities” and “urban administrative regions” should be distinguished in the studies of urban shrinkage, and special national conditions of different countries should be fully considered [41,42]. County is “city tail and township head” in Chinese urbanization system [43], which is a link between urban and rural areas with both urban and rural characteristics. Chinese county-level administrative divisions often include not only the county seat, but also some non-urban areas. If the identification of shrinking cities is not differentiated, the identification result may be expanded, “shrinking areas” rather than “shrinking cities” are more likely to be identified [44].

## 3 Research area, methods and data sources

### 3.1 Research area

**Fig. 1:**
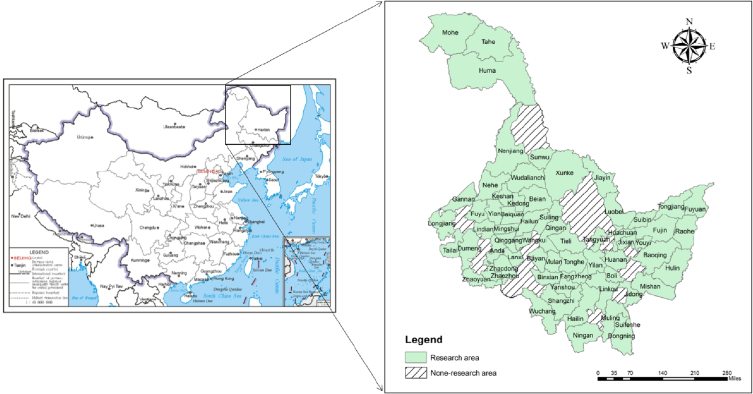
Research area. Note: The figure number is GS (2022)4315.

Heilongjiang province is located in the northeast region, which is the main commodity grain and old industrial base of China. The seventh national census results show that China's total population grew by 72.05 million between 2010 and 2020 [15]. However, the total population of Heilongjiang province decreased by about 6.46 million. The population loss of county-level is more serious, except for the counties whose data are missing due to the adjustment of administrative divisions, the average population change rate of the remaining counties is about −25.19 %, which is much lower than the provincial population change rate (-16.87 %). Heilongjiang Province is a typical research area that helps to provide reference to other provinces. In addition, in the context of deindustrialization and population loss, the shrinkage situation in Heilongjiang Province is similar to that in typical cities such as the Rust Belt in the United States, taking Heilongjiang Province as a research area helps to improve the international comparability of research results. Therefore, this paper takes 68 county-level cities except municipal districts in Heilongjiang Province as the research area, and for the timing stability of the data, it excludes part of the counties and districts that have been removing counties and establish districts or removing districts and establish counties from 2010 to 2020 (mainly concentrated in Yichun),^1^ finally 63 county-level cities in Heilongjiang Province are taken as the research area (Fig. 1).

### 3.2 Research methods

#### 3.2.1 Mutation detection method

Mutation detection method was often used to extract urban built-up area boundaries. Due to the gradual weakening of the outward light of the urban built-up area and the certain diffusing nature of the nighttime light, the intensity of the nighttime light inside the city is usually higher and shows a decreasing trend to the edge area. Therefore, it needs to set a certain threshold value for urban nighttime light. With the continuous increase of the threshold value, the light area and its perimeter will gradually shrink. When the threshold value is raised to a certain extent, the light intensity reaches the range of the built-up area of the city, and the light area will be broken from a complete polygon, and its perimeter will increase. At this time, the threshold value is to extract the light value of the built-up area of the city [45]. In this paper, the built-up area boundary data are mainly derived from the “Multi-temporal high-resolution Global City Boundary Dataset” [46]. Due to the missing value of the built-up area boundaries of Huma County, Tahe County, Mohe City, Fuyuan City, Raohe County and Xunke County in the data set, the mutation detection method was used to extract the boundaries of the above counties and cities.^2^

#### 3.2.2 Nighttime light index

Drawing on existing studies [47]，, the nighttime light index, i.e., the regional average nighttime light intensity I, is used to identify urban shrinkage. It refers to the ratio of phase elements with radiation values higher than 0 to the total number of phase elements in this region.The formula is as follows (Eq. (1)):

(1) $$I_{j}=\frac{\sum_{i=1}^{n}{\text{DN}}_{\text{ij}}}{n_{j}}$$

where: i represents the ith phase element, j represents the jth region, n represents the number of phase elements in the region, and DN_ij_ is the radiation value of the ith phase element in the jth region.

#### 3.2.3 Identification methods for urban shrinkage

This paper draws on the “two-step diagnostic method” of shrinking cities [16] to initially identify urban shrinkage based on the average annual population change rate of each city. The cities whose average population change rate is less than 0 from 2010 to 2020 are preliminarily identified as urban shrinkage. The formula is as follows (Eq. (2)):

(2) Si=∑t=2010t=2020(Pit+1−PitPit)10

where S_i_ represents the average population change rate of the ith city, t is the year, and P_it_ represents the total population of the ith city in the t year.

However, the second step is different. The second step in the traditional two-step diagnostic method is to identify the shrinking city with the evaluation index system. This paper applies nighttime lighting data to two-step diagnostics. The second step is to identify the urban shrinkage based on L, the sum of the changes of the annual nighttime light value of each city. When L is less than 0, it is identified as urban shrinkage. The formula is as follows (Eq. (3)):

(3) $$L_{\text{nj}}=\sum_{n=2010}^{n}\left(I_{\left(n+1\right)j}-I_{nj}\right)$$

L_nj_ represents the shrinkage degree of the jth city in the nth year, and I_nj_ represents the average nighttime light intensity of the jth city in the nth year.

#### 3.2.4 Spatial autocorrelation

Moran's I index was used to analyze the spatial agglomeration characteristics of county-level urban shrinkage in Heilongjiang Province, and Getis-Ord Gi* index was used to explore the hot and cold spots of urban shrinkage and reveal their spatial characteristics. Moran's I index formula is as follows (Eq. (4)):

(4) $${\text{Moran}}^{\prime }s\;I=\frac{\sum_{i=1}^{n}\sum_{j\neq 1}^{n}\omega_{\text{ij}}\left(x_{i}-\bar{x}\right)\left(x_{j}-\bar{x}\right)}{(\sum_{i=1}^{n}\sum_{j\neq 1}^{n}\omega_{ij})\sum_{i=1}^{n}{\left(x_{i}-\bar{x}\right)}^{2}}$$

where n is the number of spatial units, xi and xj are the degree of urban shrinkage of regions i and j, ‾x indicates the extent of urban shrinkage in the region. ω_ij_ is the spatial weight matrix.

When the Z score in the Moran's results is greater than or less than 1.96 and the P value is less than 0.05. The shrinking cities are clustered at 95 % confidence intervals. At the same time, the Getis-Ord Gi* index can also indentify regions of spatial aggregation. The formula is as follows (Eq. (5)):

(5) $$G_{i}^{*}=\frac{\sum_{j=1}^{n}\omega_{ij}x_{j}}{\sum_{i=1}^{n}x_{i}}$$

The meaning of each symbol in the formula is as above.

#### 3.2.5 Random effects models

Taking 63 county-level cities in Heilongjiang as the research area, this paper analyzes the variables affecting the shrinkage of county-level cities in Heilongjiang province from 2010 to 2020. Analyze select panel data, so model as follows (Eq. (6)):

(6) $$Y_{\text{it}}=\beta_{n}x_{\text{nit}}+\alpha_{i}+\varepsilon_{\text{it}}$$

here i means county and t means year, Y_it_ is the district average nighttime light intensity for the t year of the i county. X_nit_ represents the n variable of the t year of the i county. β_n_ is the coefficient of the n-th variable, ɑ_i_ represents the influencing factors that the i-th individual does not change over time. The ε_it_ is a random disturbance term for the t-year of the i-county. When ɑ_i_ are seen as random factors, the model is a random effect model.

### 3.3 Source of data

The nighttime light data is derived from the cross-sensor-calibrated “NPP-VIIRS-like NTL Data” from the Harvard Dataverse platform with a spatial resolution of 500 m and a preprocessed coordinate system of WGS_1984 [19]. The vector data of county administrative divisions in Heilongjiang Province are derived from the National Geographic Information Resources Directory Service System. The city boundary data comes from the “Multi-temporal high-resolution Global City Boundary Dataset” [46] by Gong Peng's team at Tsinghua University. The statistical data are mainly collected from Heilongjiang Statistical Yearbook, China County Statistical Yearbook (Volume of Counties and Cities), national economic and social development statistical bulletins of counties and cities, government work reports and the 14th Five-Year Plan.

## 4 Results

### 4.1 Identification results of county-level urban shrinkage in Heilongjiang Province

**Fig. 2:**
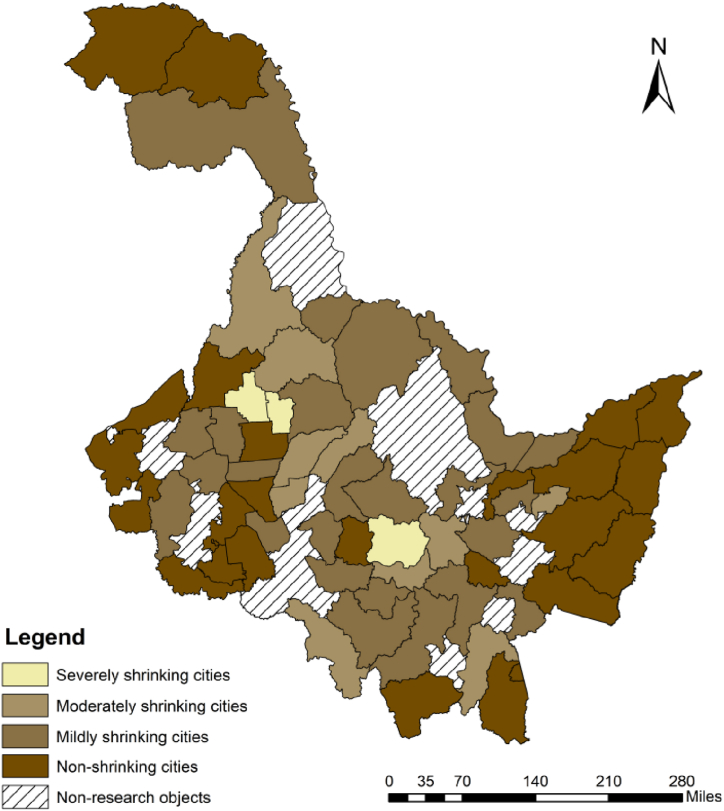
Distribution of shrinking cities in counties of Heilongjiang Province from 2010 to 2020.

In the 63 cities within the research area, the first step preliminarily identified 60 shrinking cities except Fuyuan City, Suifenhe City and Tahe County based on the rate of population change. 38 cities, accounting for 60.3 %, were finally identified by the change of average nighttime light intensity from 2010 to 2020. According to the size of Lij, the shrinking cities were divided into four categories by natural breakpoint method: severely shrinking cities (−1.72 to −1.47), moderately shrinking cities (−1.47 to −0.4), mildly shrinking cities (−0.4 to −0.01) and non-shrinking (Fig. 2).

### 4.2 Spatial characteristics of county-level urban shrinkage in Heilongjiang Province

The Moran's I index of county level urban shrinkage level in Heilongjiang Province from 2010 to 2020 is 0.07 (z-score = 2.17, p < 0.05), indicating that there are obvious spatial correlation characteristics of county level urban shrinkage in Heilongjiang Province.

**Fig. 3:**
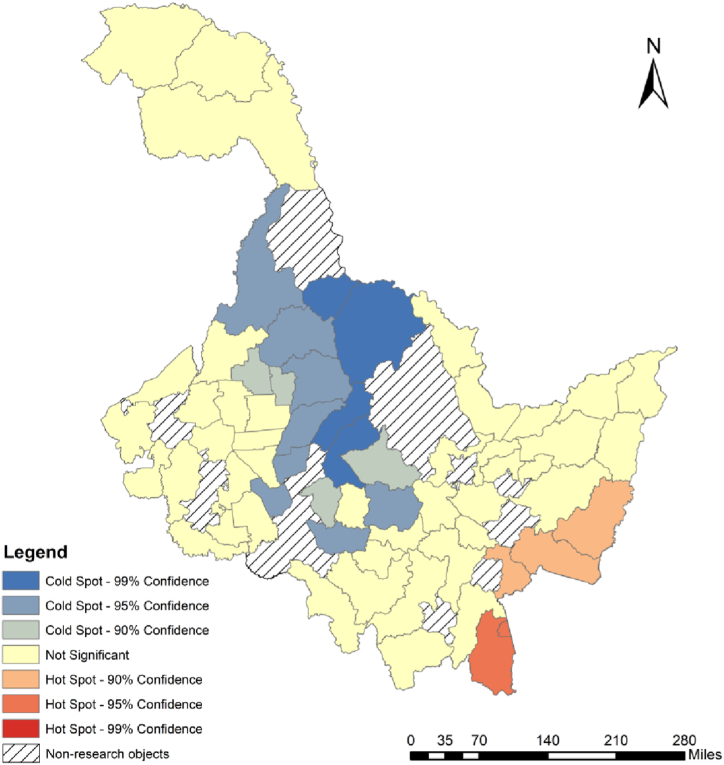
The result of Getis-Ord Gi* analysis.

In order to explore the spatial agglomeration relationship of urban shrinkage degree, Getis-Ord Gi* index was used to analyze the hot and cold spots of urban shrinkage degree (Fig. 3). The results show that hot spots are concentrated in Suifenhe, Dongning, Jidong, Mishan and other counties. Combined with Fig. 2, it can be found that non-shrinking cities in Heilongjiang Province are mainly distributed in the northeast and southwest directions. The non-shrinking cities in the northeast are concentrated around the Sanjiang Plain, which is an important commodity grain base in Heilongjiang Province, and Jiansanjiang is the most important autumn grain base in Heilongjiang Province. Every year, a large number of farmers from all over the province go to Sanjiang Plain to engage in agricultural production activities. In addition, Sanjiang Plain covers a vast area, and in the process of agricultural production, many farmers will also go to Sanjiang for temporary work during the sowing and autumn harvest seasons, thus stimulating consumption in neighboring cities and fostering urban development. In addition, the non-shrinking cities in the east are mainly distributed around the major cities in the northeast and eastern economic belt, and the non-shrinking cities in the southwest are mainly distributed around the Hadaqi Economic Belt. It can be seen that the major cities in Heilongjiang Province have a certain driving effect on the surrounding counties. At the same time, China, as the world's largest trading country of goods [48], Heilongjiang Province has also promoted economic growth through trade with Russia, especially in the northern border cities adjacent to Russia, which have shrank less than the mainland cities.The cold spots is concentrated in Xunke County, Sunwu County, Suiling County and other counties from the Greater Khingan Mountains to the central part of Heilongjiang Province. These areas have weak external ties and lack the radiation-driven effect of major cities. Therefore, the distribution of shrinking cities is more concentrated and presents the characteristics of space aggregation.

### 4.3 Time characteristics of county-level urban shrinkage in Heilongjiang Province

**Fig. 4:**
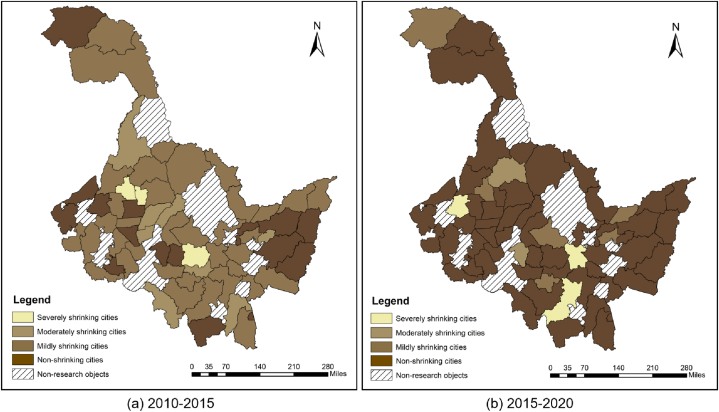
Distribution of shrinking cities in counties of Heilongjiang Province from 2010 to 2015、2015to2020.

With the year 2015 as the cut-off point, the shrinking cities of two cycles from 2010 to 2015 and 2015 to 2020 were identified respectively. In the two cycles, the variation of nighttime light intensity was calculated based on the year 2010 and 2015 respectively, and graded according to the shrinkage degree by natural breakpoint method. From 2010 to 2014, -3.92 to −3.43 were severely shrinking cities, −3.43 to −0.98 were moderately shrinking cities, and −0.98 to −0.01 were mildly shrinking cities (Fig. 4a). From 2015 to 2020, L values ranging from −0.62 to −0.46 were defined as heavily shrinking cities, from −0.46 to −0.27 as moderately shrinking cities, and from −0.27 to −0.007 as mildly shrinking cities (Fig. 4b).

**Fig. 5:**
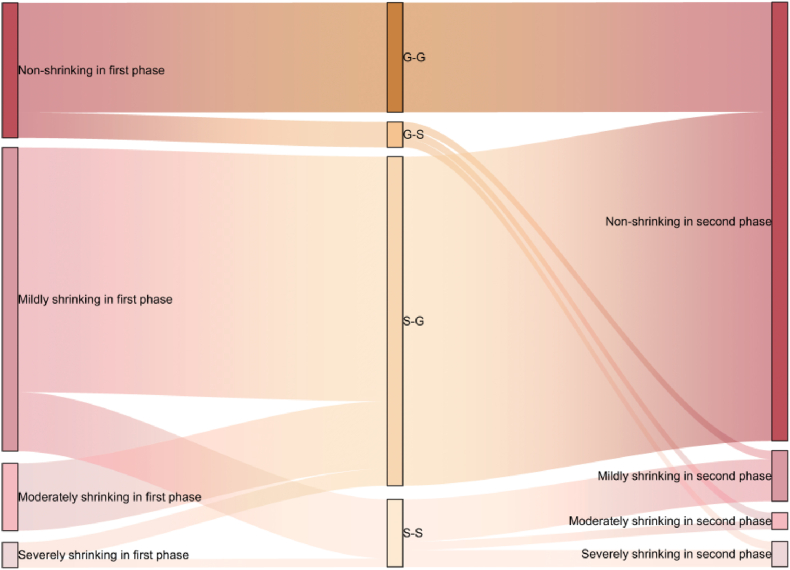
Changes in shrinkage Sankey diagram.

Between 2010 and 2015, there were 47 shrinking cities in Heilongjiang Province, accounting for 74.6 %, while between 2015 and 2020 there were 11 shrinking cities, accounting for 17.4 %. The number of shrinking cities has decreased significantly. Cities that experienced non-shrinkage in both the first and second phases are classified as G-G type cities. Cities that experienced non-shrinkage in the first phase but shrinkage in the second phase are classified as G-S type cities. Cities that experienced shrinkage in the first phase but non-shrinkage in the second phase are classified as S-G type cities. Cities that experienced shrinkage in both the first and second phases are classified as S–S type cities.Based on this, Sankey diagram is drawn (Fig. 5). As shown in the diagram, all moderately shrinking cities in the first phase transitioned to non-shrinking cities in the second phase. Only a small portion of non-shrinking cities, mildly shrinking cities, and severely shrinking cities from the first phase converted into shrinking cities in the second phase. This resulted in a significant increase in the proportion of non-shrinking cities in the second phase.

**Fig. 6:**
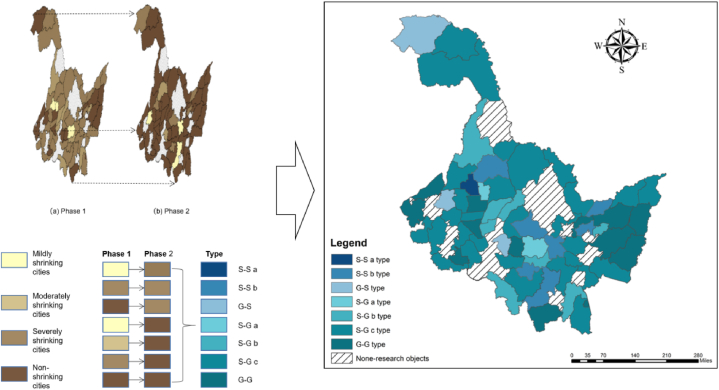
Classification of time characteristics of county-level shrinking cities in Heilongjiang Province.

And most of the severely and moderately shrinking cities in the first phase have weakened. All shrinking cities in the second phase are mildly shrinking cities (−0.98 to −0.01) if classified by the classification criteria for the degree of shrinkage in the first phase. In order to more intuitively reflect the shrinkage characteristics of Heilongjiang province counties in two phases, Further subdivision of these four types of cities based on the degree of shrinkage is performed. (Table 1), visualization results are shown in Fig. 6. Of the 63 county-level cities in the research area, 39 are of type S-G, accounting for 61.9 %, while only 3 are of type G-S. G-S type cities and S–S type cities are both mildly shrinking cities in the second phase, among them, Keshan county is S–S a type city, shrinkage degree from severe to mild. 29 S-G type cities were S-G c type cities, making up 74.35 % of the S-G type cities and 55.76 % of the non-shrinking cities in the second phase. Most of the mildly shrinking cities in the first phase experienced growth in the second phase. Only seven mildly shrinking cities are still shrinking. Non-shrinking cities in the second phase are also grown mainly by mildly shrinking cities. It can be seen that the shrinkage of county-level cities in Heilongjiang Province occurred mainly in the first half between 2010 and 2020. Between 2015 and 2020, most of the mildly shrinking cities regained growth and became non-shrinking cities, with eight moderately shrinking cities achieving growth and the weakening of the shrinkage degree as severely shrinking cities transformed into non-shrinking and mildly shrinking cities. Only a few cities shrank during this phase, and the shrinkage was weak.

**Table 1:** All types of cities.

Type of city		Basis of division		Counties	Number	Proportion
		First phase	Second Phase			
G-G type cities		Non-shrinking	Non-shrinking	Mulan County, Longjiang County, Gannan County, Hulin City, Baiquan County, Baoqing County, Raohe County, Zhaozhou County, Huachuan County, Fujin City, Suifenhe City, Ning ‘an City, Qinggang County	13	20.63 %
G-S type cities		Non-shrinking	mildly shrinking	Bayan County, Fuyu County, Mohe City	3	4.76 %
S-G type cities	S-G a type	severely shrinking	Non-shrinking	Tonghe County, Kedong County	2	3.17 %
	S-G b type	moderately shrinking	Non-shrinking	Fangzheng County, Wuchang City, Youyi County, Muling City, Nenjiang County, Hailun City, Wangkui County, Suiling County	8	12.69 %
	S-G c type	mildly shrinking	Non-shrinking	Bin County, Shangzhi City, Yi ‘an County, Tailai County, Nehe City, Jidong County, Mishan County, Luobei County, Zhaoyuan County, Lindian County, Dumeng Autonomous County, Jiayin County, Huannan County, Tangyuan County, Fuyuan City, Tongjiang City, Boli County, Dongning County, Linkou County, Bei ‘an City, Xunke County, Sunwu County, Anda City, Zhaodong City, Lanxi County, Qing ‘an County, Mingshui County, Huma County, Tahe County	29	46.03 %
S–S type cities	S–S a type	severely shrinking	mildly shrinking	Keshan County	1	1.58 %
	S–S b type	mildly shrinking	mildly shrinking	Yilan County, Yanshou County, Suibin County, Jixian County, Tieli City, Hailin City, Wudalianchi City	7	11.11 %

**Fig. 7:**
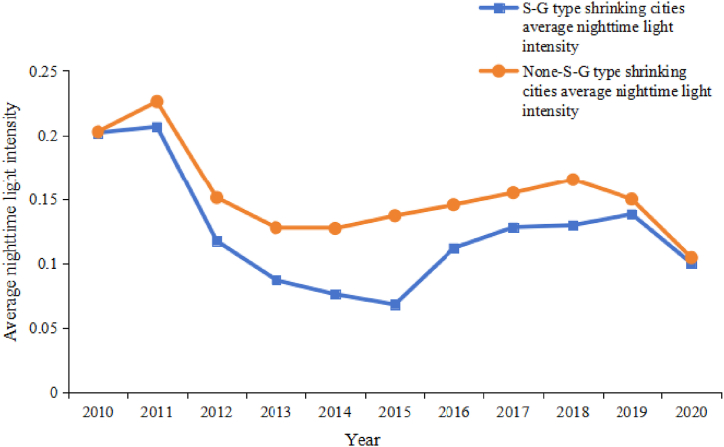
Change map of average nighttime light intensity in S-G and none-S-G type shrinking cities.

But 28 of the 39 S-G cities were still identified as shrinking cities between 2010 and 2020, accounting for 71.7 % of S-G cities and 73.6 % of all shrinking cities. The average value of nighttime light of 28 S-G shrinking cities and 10 non-S-G shrinking cities in each year was calculated to draw a line graph (Fig. 7). From 2010 to 2015, the light value of S-G shrinking city decreased year by year after a small increase, and then increased again with 2015 as the inflection point, and maintained a small increase from 2016 to 2019. But the increase after 2015 was much smaller than the decline in the previous phase. For non-S-G shrinking cities, the average nighttime light intensity continued to decline between 2011 and 2012, maintained a small increase after 2013, and began to decline after reaching the peak in 2018.

In summary, although the urban shrinkage in Heilongjiang Province is concentrated [40], the shrinkage at the county level has not continued., but have the characteristics of “great recession, small growth.” Between 2011 and 2013, the intensity of nighttime lights in both shrinking cities decreased significantly, but after 2015, the intensity of nighttime lights in shrinking cities recovered slightly. Given the impact of COVID-19, the intensity of nighttime lights in shrinking cities decreased in 2020, but the growth trend should not be overlooked. For the county area of Heilongjiang Province, although the total population decreased significantly in the statistics, the intensity of economic activity in the county did not decreased continuously. On the one hand, the loss of districts may result in a comparatively high proportion of the agricultural population. On the other hand, each county may have more agricultural population in-situ urbanization, promote county agglomeration development.

### 4.4 The theoretical analysis of factors influencing urban shrinkage

**Fig. 8:**
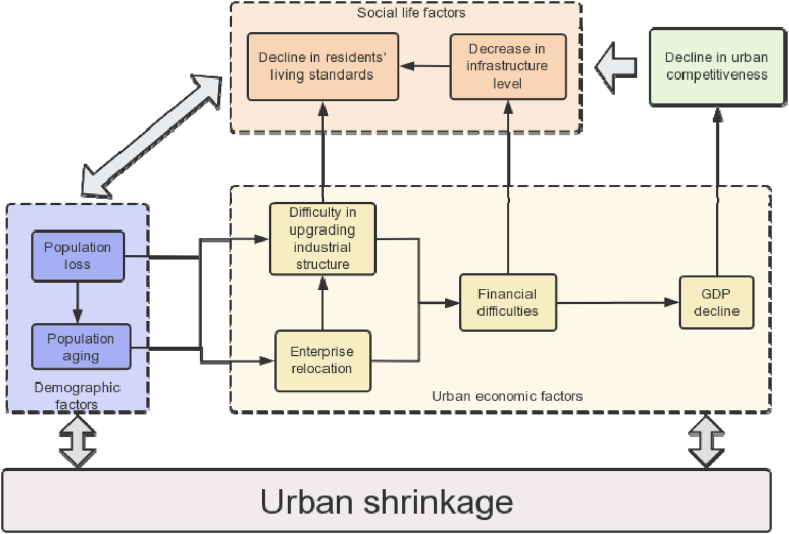
Theoretical framework diagram of factors influencing urban shrinkage.

The core characteristic of urban shrinkage is population outflow, with the departing population often being the young and productive labor force. Consequently, as population outflow occurs, the urban population structure gradually ages. This leads to an insufficient supply of labor in the urban labor market, causing some factories and businesses to choose to relocate. The changes in businesses and population pose difficulties for the upgrading of the city's industrial structure and also disrupt the balance of the city's fiscal revenue and expenditure, resulting in a decline in GDP. At this point, the economic vitality of the city starts to decline, and the reduced government revenue makes it difficult to maintain urban infrastructure, leading to a decline in the level of infrastructure. Simultaneously, the decrease in the number of businesses and the challenges in upgrading the industrial structure lead to a reduction in employment opportunities, resulting in a decline in the living standards of city residents. During this time, the city often struggles to maintain a favorable position in urban competition, leading to population outflow and further promoting population loss, which affects urban shrinkage. Furthermore, urban shrinkage in turn stimulates population outflow and economic decline, creating a cycle of repetitive processes. The theoretical analytical framework is illustrated in Fig. 8.

### 4.5 Influencing factors of urban shrinkage in county level of Heilongjiang Province

Taking the average nighttime light intensity of each city from 2010 to 2020 as the dependent variable. Reference to existing research [49,50] and combined with the theoretical framework, variables selection is shown in Table 2. In order to explore the heterogeneity of variables affecting shrinking cities and non-shrinking cities, Model 1 and Model 2 were constructed with the samples of shrinking cities and non-shrinking cities from 2010 to 2020 respectively. Since both models used panel data, Hausmann test is required to select models. The Hausman test result of model 1 is P = 0.0999 > 0.05, and the Hausman test result of model 2 is P = 0.4077 > 0.05. Therefore, random effects model is used to construct Model 1 and Model 2. In order to eliminate the dimensional influence of each variable, the range method was used to normalize the data before regression.

**Table 2:** The main explanatory variables and their meanings.

Criterion layer	Index layer	Index calculation
Demographic factors	Population density	Total population/administrative area
	The proportion of primary and secondary school students	Total number of primary and secondary students/Total population
Urban economic factors	GDP per capita	GDP/Total population
	The number of industrial enterprises above designated size	
	Financial revenue and expenditure	Fiscal revenue/expenditure
	The proportion of secondary and tertiary production	Total output value of secondary and tertiary industries/GDP
Social life factors	household savings balance	
	highway mileage	

The regression results of the models are shown in Table 3. From the perspective of population factors, population density has a significant positive impact on non-shrinking cities, while the impact on shrinking cities is not significant. On the one hand, this may be because most of the population lost by shrinking cities are high-level labor force [51]. When the city shrinks to a certain level, the population left behind in the city is usually the elderly or those who lack the competitive ability to work outside, and these populations have limited contribution ability to urban economic development. In this context, the change of the total population in a city is difficult to reflect the status of urban development. On the other hand, from the perspective of data accessibility, the population data chosen for this paper is the total population of counties, and the changes of total population can, to some degree, reflect changes in the population of cities. However, the phenomenon of “hollow villages” in China is getting more serious [52], and the loss of rural population may also contribute to the decline of total population. Therefore, for shrinking cities, the effect of population density is not significant. For non-shrinking cities, there is no interference from these two factors. The flow of people into non-shrinking cities tends to be more capable of working and, as the total population increases, it can exclude the disturbance of rural emigration. Therefore, for non-shrinking cities, population density has a significant positive impact on the level of urban development. The proportion of primary and secondary school students can reflect the vitality of a city's population structure. The younger the urban population structure, the better it can ensure the supply of labor force, thereby promoting urban development. In Model 2, the coefficient of the proportion of primary and secondary school students is positive, which proved that the proportion of primary and secondary school students in a shrinking city can promote urban development and slow down urban shrinkage to some extent.

**Table 3:** Model regression results.

Independent variable	Model1	Model2
	non-shrinking cities	shrinking cities
Household savings balance	0.291***	0.119**
The number of industrial enterprises above designated size	−0.425***	−0.047
GDP per capita	0.268***	−0.736***
Financial revenue and expenditure	−0.074*	0.119***
Highway mileage	0.282***	−0.007
The proportion of primary and secondary school students	0.064	0.114**
The proportion of secondary and tertiary production	0.117**	0.117**
Population density	1.211**	−0.101
Chi2	102.38	76.55
P > Chi2	0	0
R2	0.2517	0.1574
Total sample size	275	418

Note: *, ** and *** respectively represent passing the significance test underthe confidence interval of 90 %, 95 % and 99 %.

In terms of social factors, household saving balance has a significant positive impact on both non-shrinking cities and shrinking cities. Household saving balance reflects the wealth accumulation level of urban resident and can the standard of living and employment of urban residents to a certain extent. When urban resident can obtain sufficient material returns through work and other ways in the city, It often does not choose to flow out to other cities, thus promoting the development of cities. Highway length has a significant positive effect on non-shrinking cities, which reflects the promoting effect of traffic conditions on population flow. For non-shrinking cities, better transport means they can absorb more migrants and thus boost economic growth. In addition, highway length often reflects the high level of county infrastructure construction, thus promoting the growth of non-shrinking cities.

In terms of economic factors, the number of industrial enterprises above designated size has a significant negative impact on non-shrinking cities, which may be related to the development history of Heilongjiang Province. Heilongjiang Province had a comparatively strong industrial base in the early years of the China. After the reform and opening up, Heilongjiang Province was unable to achieve the timely transformation of its industrial structure, and factories in counties were often unable to absorb high-level labor force, young people were also frequently reluctant to work in industrial businesses [53], which resulted in the outflow of population. At the same time, in the process of urbanization, the development of industrial enterprises promotes economic growth, but inevitably leads to environmental pollution [54]. And China's relatively low energy efficiency tends to contribute to increased pollution [55,56]. Due to the large environmental pollution caused by the production of industrial enterprises, given the population loss exodus brought on by the pursuit of high-quality living standards, it is difficult for cities with a large number of industrial enterprises to hold a dominant position in urban competition [57]. For the shrinking cities and the non-shrinking cities, the fiscal expenditure has the opposite effect on them. In shrinking cities, the coefficient of financial revenue and expenditure is positive, which means that the proportion of financial revenue and expenditure can promote the development of the shrinking cities. In order to achieve “smaller and better development” for shrinking cities, focus should be placed on enhancing the government's financial capability and effectively combining available resources [58]. For non-shrinking cities, the more complete the infrastructure is, the better it can improve its competitiveness in urban competition and absorb more migrants, which often requires more financial input, so the impact of financial revenue and expenditure is negative. The coefficients of the proportion of the secondary and tertiary industries in shrinking cities and non-shrinking cities are both positive, which means that it can have a significant positive impact on the development of the two types of cities. The proportion of secondary and tertiary industries can reflect the advanced degree of urban industrial structure. For shrinking cities, the more advanced the industrial structure is, the more it can reduce the degree of shrinkage. In Model 1 and Model 2, per capita GDP has a completely opposite effect on shrinking cities and non-shrinking cities. This indicates that the county-level shrinking cities in Heilongjiang Province from 2010 to 2020 are still in the early stage of urban shrinkage. The city's economy is still expanding at this stage, and it is in the stage between the inflection point of population loss and economic growth, despite the loss of urban population, and the decrease of economic activities and vitality [17]. Therefore, per capita GDP has a significant negative impact on shrinking cities.

### 4.6 Robustness test of the model

In order to verify the robustness of the model in the influencing factors analysis, Model 1 and Model 2 were tested by gradually increasing variables. Among them, the number of medical beds can reflect the medical level of the city. According to existing studies, the population loss of resource-exhausted cities is more serious [59]. Therefore, for the counties and cities under the authority of the counties and cities that are resource-exhausted cities or not, dummy variables are created. And they are successively included in the regression model. The results are shown in Table 4.

**Table 4:** The results of the robustness test.

Independent variable	Model1	Model2	Model3	Model4	Model5	Model6
	non-shrinking cities	shrinking cities	non-shrinking cities	shrinking cities	non-shrinking cities	shrinking cities
Household savings balance	0.291***	0.119**	0.298***	0.124**	0.299***	0.121**
The number of industrial enterprises above designated size	−0.425***	−0.047	−0.425***	−0.049	−0.424***	−0.052
GDP per capita	0.268***	−0.736***	0.264***	−0.729***	0.263***	−0.702***
Financial revenue and expenditure	−0.074*	0.119***	−0.074*	0.121***	−0.075*	0.122***
Highway mileage	0.282***	−0.007	0.285***	0.011	0.291***	−0.01
The proportion of primary and secondary school students	0.064	0.114**	0.061	0.112**	0.065	0.111**
The proportion of secondary and tertiary production	0.117**	0.117**	0.114*	0.119**	0.113*	0.099**
Population density	1.211**	−0.101	1.256**	−0.104	1.360**	−0.109
Number of medical beds			−0.009	−0.015	−0.006	−0.017
Whether under a resource-exhausted city					0.021	−0.041
Chi2	102.38	76.55	101.43	76.57	100.95	78.83
P > Chi2	0	0	0	0	0	0
R2	0.2517	0.1574	0.2529	0.1574	0.2545	0.1584
Total sample size	275	418	275	418	275	418

Note: *, ** and *** respectively represent passing the significance test underthe confidence interval of 90 %, 95 % and 99 %.

In Table 4, after adding variables successively, the coefficient of the proportion of secondary and tertiary industries in Model 6 changes slightly compared with model 2 and model 4, but the significance and positive or negative directions do not change significantly. In addition, there is no significant change in the significance and coefficient values of each influencing factor between non-shrinking cities and shrinking cities, indicating that the influencing factor models has a high robustness.

## 5 Discussion

Based on the “two-step diagnosis method” of shrinking cities, this paper uses cross-sensor-corrected “NPP-VIIRS-like NTL Data” to identify the urban shrinkage of county-level cities in Heilongjiang Province from 2010 to 2020 from the perspective of physical cities. Moran's I index and Getis-Ord Gi* index were used to reveal its spatial characteristics. Taking 2015 as the boundary, the temporal characteristics of county-level urban shrinkage in Heilongjiang Province were analyzed, and variables were selected from the three dimensions of population, urban economy and social life to explore the influencing factors of county-level urban shrinkage in Heilongjiang Province. The results shown that the county level cities in Heilongjiang Province have the characteristics of spatial agglomeration and “big recession, small growth” in time. It is also found that the shrinkage of county level cities in Heilongjiang Province from 2010 to 2020 is mainly in the stage between the two turnning points of population loss and economic recession. To deal with the phenomenon of urban shrinkage, we should begin by focusing on ways to enhancing inhabitants' quality of life, enhancing the governance ability of urban government, upgrading the industrial structure and adjusting the population structure.

In the study, when the “two-step diagnostic method” was used to identify urban shrinkage, it was found that the identification results of the first step were significantly different from those of the second step. The shrinking cities identified by a single population index were significantly more than those identified by nighttime light intensity, and from the perspective of time characteristics, the decrease of nighttime light intensity lagged the population loss, while some of the cities with the decrease of nighttime light intensity did not have economic recession. This further proved the conclusion that there is a lag between the turnning point of population, economic activity intensity, economic level and spatial quality of shrinking cities. Therefore, although population loss is the reason for the decline in the intensity of urban economic activity, it is may not comprehensive to identify shrinking cities only from population change. In addition, this paper identified the county-level shrinking cities in Heilongjiang Province from the perspective of physical cities. The identification results based on the nighttime lights of urban built-up areas exclude the interference of non-urban areas and have stronger reality.

## 6 Conclusions

### 6.1 The characteristics of spatial aggregation exist in county-level shrinking cities in Heilongjiang Province

Within the research area, there are significant spatial aggregation characteristics of each shrinking city. Shrinking cities are concentrated in Xunke County, Suiling County and Qingan County, interleaved in the hinterland of Heilongjiang Province. Non-shrinking cities are mainly distributed in Hulin City, Dongning City and Suifenhe City, which are located around the Sanjiang Plain and major cities in the Northeast Eastern Economic Belt, and a large number of non-shrinking cities gather around the Hadaqi Economic Belt. It can be seen that agricultural production activities and major cities in Heilongjiang Province can promote the development of surrounding counties.

### 6.2 The shrinkage of county-level cities in Heilongjiang Province from 2010 to 2020 showed the characteristics of “big recession and small growth"

Taking 2015 as the boundary, the counties of Heilongjiang Province mainly shrunk in the previous phase, and the degree of shrinkage was more serious. However, after 2015, most counties are showing signs of re-growth. Therefore, more active and effective measures should be taken for the management of county-level urban shrinkage in Heilongjiang Province to cultivate the county economy vigorously and inject vitality into the economic development of counties.

### 6.3 The shrinkage of county-level cities in Heilongjiang Province is still in the middle stage of two turnning points of population loss and economic recession

Heilongjiang's shrinking cities are still mainly in the stage of GDP growth. According to existing studies, in the process of urban shrinkage, population loss, weakening of economic activity and recession will occur in turn. Nighttime lights reflect the intensity of economic activity in cities, and their decline tends to lag behind the decline in urban population. Among the influencing factors, per capita GDP has a negative impact on shrinking cities, which is proved the shrinkage of county-level cities in Heilongjiang Province is still in the middle stage between the turnning point of population loss and economic recession. At this time, the process of urban shrinkage is often still reversible, more active and effective measures should be adopted to take appropriate governance measures for some shrinking cities.

### 6.4 The living quality of residents, the vitality of urban population and the vitality of urban economy can have a significant impact on urban shrinkage

Population loss is the first step in urban shrinkage. Population tends to relocate in pursuit of a higher quality of life. Based on the above research findings, wealth accumulation of urban residents will promote the development of shrinking cities, and the proportion of urban young population will also have a promoting effect. For the urban economic conditions, the advanced degree of the industrial structure and financial status will have an impact on the urban shrinkage. The more advanced the industrial structure and the larger the proportion of financial revenue and expenditure, the more it can promote the development of shrinking cities.

### 6.5 Policy recommendations

#### 6.5.1 Implement differentiated urban development strategies based on urban location, pillar industries etc

The county-level shrinking cities in Heilongjiang Province are in the early stage of shrinkage, combined with urban characteristics to explore differentiated development strategies, still help to shrinking city to achieve re-growth. For Heilongjiang Province, agricultural production activities and the major cities around the county can promote its urban development. For the county around the northeast Sanjiang Plain, the goal should be to achieve agricultural mechanization and modernization, thus promoting the development of the county; Hadaqi economic belt, the east economic belt in northeast of the major cities surrounding the county, should pay attention to be linked with the neighboring big cities, to achieve industrial complementarity and win-win cooperation.

#### 6.5.2 Improve government revenue capacity and concentrate resources to achieve smart growth

The shrinking county government of Heilongjiang Province should pay attention to realizing the development of “smaller and smarter growth,” get rid of the traditional lightweight growth model, properly concentrate public resources, improve urban infrastructure, and the quality of life of residents while ensuring good financial functioning.

#### 6.5.3 Actively promote the new type of urbanization with the county city as the important carrier, and play the unique advantage of the county city to carry the rural population transfer

Rural population loss in Heilongjiang Province is difficult to reverse, We should focus on improving the basic education level of the county and exploring the social security system of transferring the population from agriculture into the city. Promote the transfer of agricultural population close to urbanization, open the “money、land and people” element gathering channel, to avoid population loss exacerbated urban shrinkage.

#### 6.5.4 Deepen the adjustment of the industrial structure to ensure the basic status of primary industry, while improving the efficiency of green production, optimizing the structure and quality of third industry, and promoting the integration of third industry

For county cities in Heilongjiang province, agricultural production should be ensured while extending the agricultural industrial chain and promoting deep processing of agriculture. Reduce production pollution in industrial enterprises and improve the quality of the urban environment. We will actively develop tourism resources, explore growth models such as tourism with local characteristics, enhance the advanced level of industrial structure, achieve the integration of three-industry integration, create more quality jobs, and build a new growth pole in the county.

## 7 Limitations of the study

Few limitations remain. Firstly, for data accessibility reasons, this paper extracts nighttime light data from built-up areas for the two cycles around 2015 with city boundaries in 2010 and 2015. Although urban boundary expansion is a slow process, and the closer the city center, the more stable the nighttime lights, the more accurate the results can be further improved by obtaining year-by-year city boundary data through remote sensing interpretation.

Secondly, for data timing stability, only eight variables are selected as influencing factors. Further consideration could be given to the integration of urban geography, natural characteristics and other factors to improve the comprehensiveness of the influencing factors.

Finally, the study scale is selected for only ten years, and the urban shrinkage characteristics on the longer scale can be explored.

## Data availability statement

Data will be made available on request.

## CRediT authorship contribution statement

**Junqi Huo:** Data curation, Formal analysis, Methodology, Software, Visualization, Writing – original draft. **Shanlin Huang:** Conceptualization, Funding acquisition, Methodology, Project administration, Supervision, Validation, Writing – review & editing.

## Declaration of competing interest

The authors declare that they have no known competing financial interests or personal relationships that could have appeared to influence the work reported in this paper.

## Footnotes

1 In 2015, Shuangcheng was set up as a district. In 2019, Tangwang, Fenglin, Nanchay and Daqingshan were removed from districts and set up as counties.

2 The nighttime light in the administrative division of Huma County was too weak, so the area with radiation value greater than 0 was taken as the urban boundary of Huma County in 2015.
